# Mechanical Method for Correction of Facial Expression

**Published:** 1907-06-15

**Authors:** Weston A. Price


### Mechanical Method for Correction of Facial Expression.

BY WESTON A. PRICE, D. D. S.

Dr. Price gave a lecture which is not reportable, as he talked from lantern slides and models that can not be reproduced in print. The scheme adopted by Dr. Price is to correct the mal-occlusion caused by loss of teeth, producing mal-occlusion of different kinds, and in some cases no occlusion at all. By raising the bite with crown and bridge work, he not only opens the bite, but restores the contour and occlusion, and produces excellent esthetic results. Some of the cases shown seem almost hopeless, but after treatment they show remarkable improvement in appearance and masticating powers.

